# *Staphylococcus pseudintermedius* Sbi paralogs inhibit complement and bind IgM, IgG Fc and Fab

**DOI:** 10.1371/journal.pone.0219817

**Published:** 2019-07-23

**Authors:** Alaa H. Sewid, M. Nabil Hassan, A. M. Ammar, David A. Bemis, Stephen A. Kania

**Affiliations:** 1 Department of Biomedical and Diagnostic Sciences, University of Tennessee, Knoxville, Tennessee, United States of America; 2 Department of Microbiology, Faculty of Veterinary Medicine, Zagazig University, Zagazig, Egypt; Pusan National University, REPUBLIC OF KOREA

## Abstract

The success of staphylococci as pathogens has been attributed, in part, to their ability to evade their hosts’ immune systems. Although the proteins involved in evasion have been extensively studied in staphylococci affecting humans little characterization has been done with *Staphylococcus pseudintermedius*, an important cause of pyoderma in dogs. *Staphylococcus aureus* binder of immunoglobulin (Sbi) interferes with innate immune recognition by interacting with multiple host proteins. In this study, a *S*. *pseudintermedius* gene that shares 38% similarity to *S*. *aureus Sbi* was cloned from *S*. *pseudintermedius* strains representative of major clonal lineages bearing two paralogs of the protein. Binding of immunoglobulins and Fab and Fc fragments as well as interaction with complement was measured. *S*. *pseudintermedius* Sbi protein bound IgG from multiple species and canine complement C3, neutralized complement activity and bound to canine IgM and B cells. Evidence from this work suggests Sbi may play an important role in *S*. *pseudintermedius* immune evasion.

## Introduction

*Staphylococcus pseudintermedius*, distinguished from *Staphylococcus intermedius* in 2005, is a Gram-positive, coagulase-positive bacterium commonly found on the skin, nares, mouth, pharynx, and anus of healthy dogs, cats, and horses [1]. It is one of the most common infectious agents seen in small-animal veterinary practice worldwide and causes diseases including pyoderma, otitis and urinary tract infections [2] and occasionally infects humans [3, 4]. *S*. *pseudintermedius* strains isolated from North America and Europe often have resistance to multiple classes of antimicrobials [5, 6]. Due to widespread resistance, development of additional therapeutic strategies such as vaccines is an important research priority.

**Fig 1:**
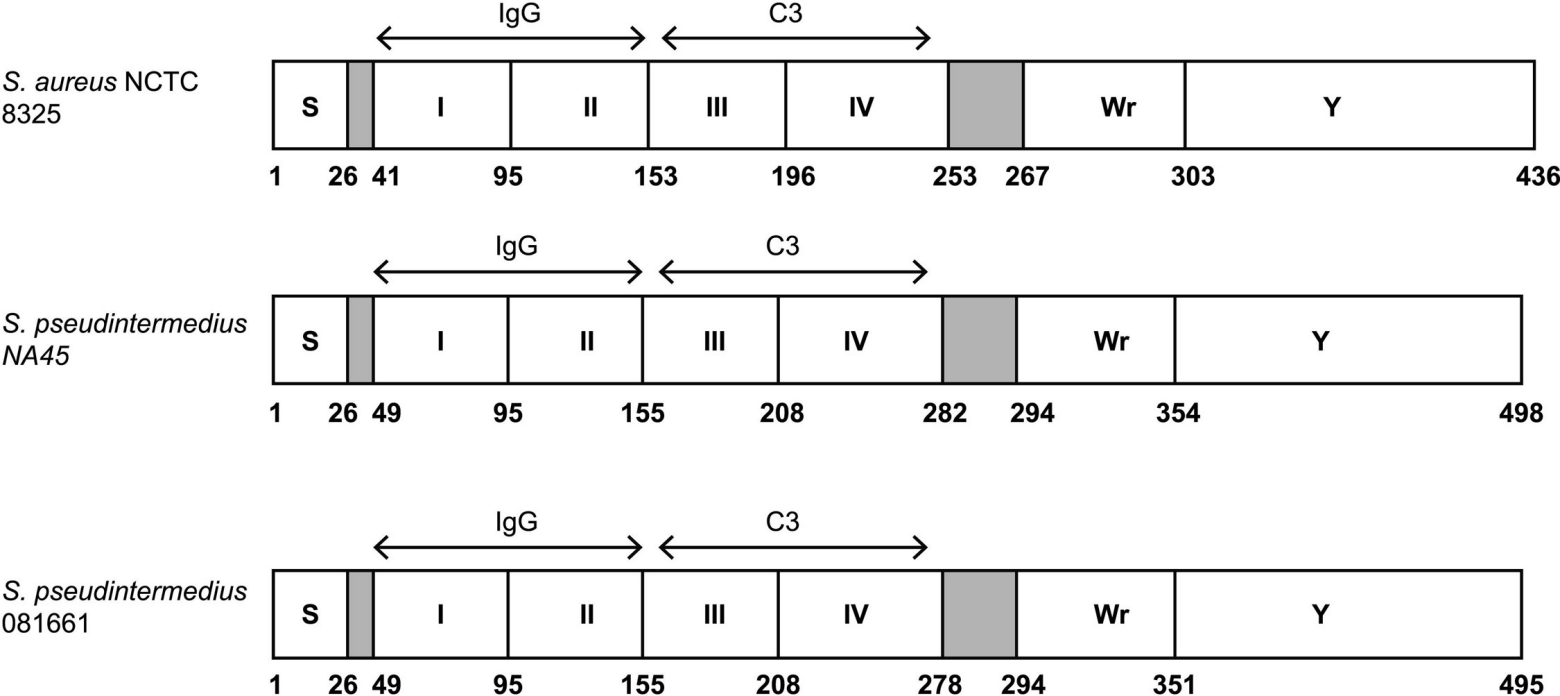
Schematic representation of *S*.*pseudintermedius* Sbi domains. The predicted structures of *S*. *pseudintermedius* Sbi proteins from strains NA45 and 081661 are depicted in comparison with *S*.*aureus* NCTC 8325 [11]. These include the signal peptides (S), N-terminal IgG-binding domains I and II (corresponding to *S*. *aureus* regions D1 and D2) and domains III and IV that correspond to complement C3 binding domains D3 and D4 of *S*. *aureus* [30]. These are followed by the Wr domain which is rich in proline and the Y domain that is tyrosine rich and likely involved in membrane binding [30]. Numbers correspond to amino acid residues.

Cell wall-associated and secreted proteins responsible for immune evasion are preferred targets for immunotherapy and prophylaxis [7]. One such protein, staphylococcal binder of immunoglobulin (Sbi), is a multifunctional bacterial protein shown in *S*.*aureus* to interfere with innate immunity by inhibiting opsonophagocytosis and complement activation [8, 9] by interacting with multiple host proteins [10, 11]. It lacks the cell wall-anchoring LPXTG motif and is non-covalently bound to the cell surface by its interaction with lipoteichoic acid and is also secreted [12]. *S*. *aureus* Sbi has been extensively studied and serves as a basis for comparison with orthologous proteins. The mature protein is composed of six regions (Fig 1). The N-terminal portion of Sbi contains two immunoglobulin binding domains (IgBDs), Sbi-I and Sbi-II (S1 Fig), that are similar to the IgBDs of staphylococcal protein A. They are triple-helical bundles that associate with the Fcγ of IgG [10]. Sbi-III and Sbi-IV domains are independently folded and interfere with the complement system by binding to host complement components C3 and Factor H and form tripartite Sbi:C3:Factor H [13]. Thus, Sbi disrupts the molecular link between innate and adaptive immune responses. It likely acts synergistically with protein A to block B cell antigen recognition by binding to the VH3 region of IgM molecules, inducing B cell apoptosis [14]. It also inhibits complement receptor 2 (CR2) interaction of complement component C3 (antigen-associated C3dg or iC3b) via Sbi III and IV domains [10, 13, 15]. Wr is associated with cell membrane-spanning and the C-terminus contains a tyrosine-rich Y region shown to be involved in IgG-mediated signal transduction [8–10]. A *S*. *pseudintermedius* gene encoding a predicted protein with 38% similarity to *S*. *aureus* Sbi is often annotated as IgG-binding protein Sbi, however, to our knowledge its functional properties have not been examined. Based on its predicted amino acid similarity to *S*. *aureus* Sbi in two apparently paralogous forms of the protein, putative *S*. *pseudintermedius* Sbi (pSbi) from two clonal lineages was studied to determine its biological properties as they relate to a potential role in the virulence of *S*. *pseudintermedius*.

## Materials and methods

### Blood and serum samples

Our experimental protocol was reviewed and approved by the University of Tennessee Institutional Animal Care and Use Committee (IACUC) for the collection of blood samples from dogs (2474–0716). Equine, feline, and bovine blood were obtained from clinically normal animals under protocol 2298–0914 and in accordance with institutional policy at the University of Tennessee College of Veterinary Medicine. Surplus sera from routine testing and control samples from other species were used in accordance with institutional policy.

### Bacterial strains

A total of 17 clinical isolates obtained from the University of Tennessee, College of Veterinary Medicine Clinical Bacteriology Laboratory as well as from European and North American collaborators through previous studies, as noted in Table 1, were used. Methods for bacterial isolation, identification and antimicrobial susceptibility testing were those routinely used in the laboratory [16, 17]. The genetic backgrounds of the isolates were determined using multilocus sequence typing (MLST) described previously [18]. Strains used in the study include representatives of sequence types from clonal complexes prevalent in the United States and Europe including CC68, CC71 and CC84 [5, 6].

**Table 1:** Origins and sequence type of *S*. *pseudintermedius* strains.

*S*.*pseudintermedius* strain	Country of Isolation	Multilocus Sequence Type	Clonal Complex	Sbi genotype	Reference
NA45	USA	ST84	CC84	I	[6]
NA16	USA	ST71	CC71	II	[6]
081661	USA	ST71	CC71	II	[6]
E140 (DK 729)	Denmark	ST71	CC71	II	[19]
063228	USA	ST68	CC68	I	[6]
08521a	USA	ST68	CC68	I	[6]
081294	USA	ST68	CC68	I	[6]
NA12	USA	ST64	CC84	I	[6]
57395	Israel	ST45	none	II	[20]
E141(ED99)	Scotland	ST25	none	I	[21]
FMV5699/07	Portugal	ST207	none	none	[22]
FMV2218-10	Portugal	ST198	none	I	[22]
FMV2183-10	Portugal	ST197	none	II	[22]
FMV9/08	Portugal	ST17	CC68	none	[22]
NA73	USA	ST143	none	none	[6]
NA74	USA	ST133	none	none	[6]
Am33	Thailand	ST111	none	II	[23]

### DNA extraction and PCR amplification

Bacteria were grown on trypticase soy agar plates with 5% sheep blood overnight at 37°C. For each sample, a single colony was suspended in 5 ml of trypticase soy broth (TSB) (Becton, Dickinson and Co.) and incubated on a rotary shaker at 225 rpm at 37°C. Bacteria were harvested and DNA extracted (UltraClean Microbial DNA Isolation Kit, Qiagen). PCR primers for amplification and sequencing were designed using an online tool (PrimerQuest, Integrated DNA Technologies), using *Sbi* gene sequences obtained from genomic data for strains NA45, 063228, and 081661 [24] with GenBank accession numbers CP016072.1, CP015626.1, and CP016073.1, respectively. PCR amplification of the full-length pSbi protein coding region was carried out using primers with restriction enzyme sites for *NcoI* and *BamH1* (Table 2). The reaction mixtures consisted of 25 μl total volume containing 2.5 μl of genomic DNA, 20 pmol of each primer (1 μl), 12.5 μl of rTaq polymerase enzyme (including dNTP mixture and buffer) and 8 μl of nuclease-free water. Amplification conditions consisted of an initial denaturation (94°C for 1.5min) followed by 35 cycles of denaturation (94°C for 60s), annealing (55°C for 2min), and extension (72°C for 60 s), with a single final extension (72°C for 5 min).

**Table 2:** PCR primers.

Primer sequence (5'-3')	
Forward	F(NA45);TGTCACCATGGAAGAAACAGAAGGGAATAAACAA F(063228, 081661);TTTGGCCATGGATGAAAACTAAATACACAGC
Reverse	R(NA45);TGTCAGGATCCTTGAAGAAAGAGAAAAGATTG R(063228); TTTGGGGATCCTTGA AGA AAGATAAGA AACCA R(081661); TTTGGGGATCCTTGAAGAAAGAGAAGATACTG

Restriction enzyme sites (underlined) for *NcoI* and *BamH1* were included in the forward primer and reverse primers.

### *pSbi* sequence analysis and secondary structure

**Fig 2:**
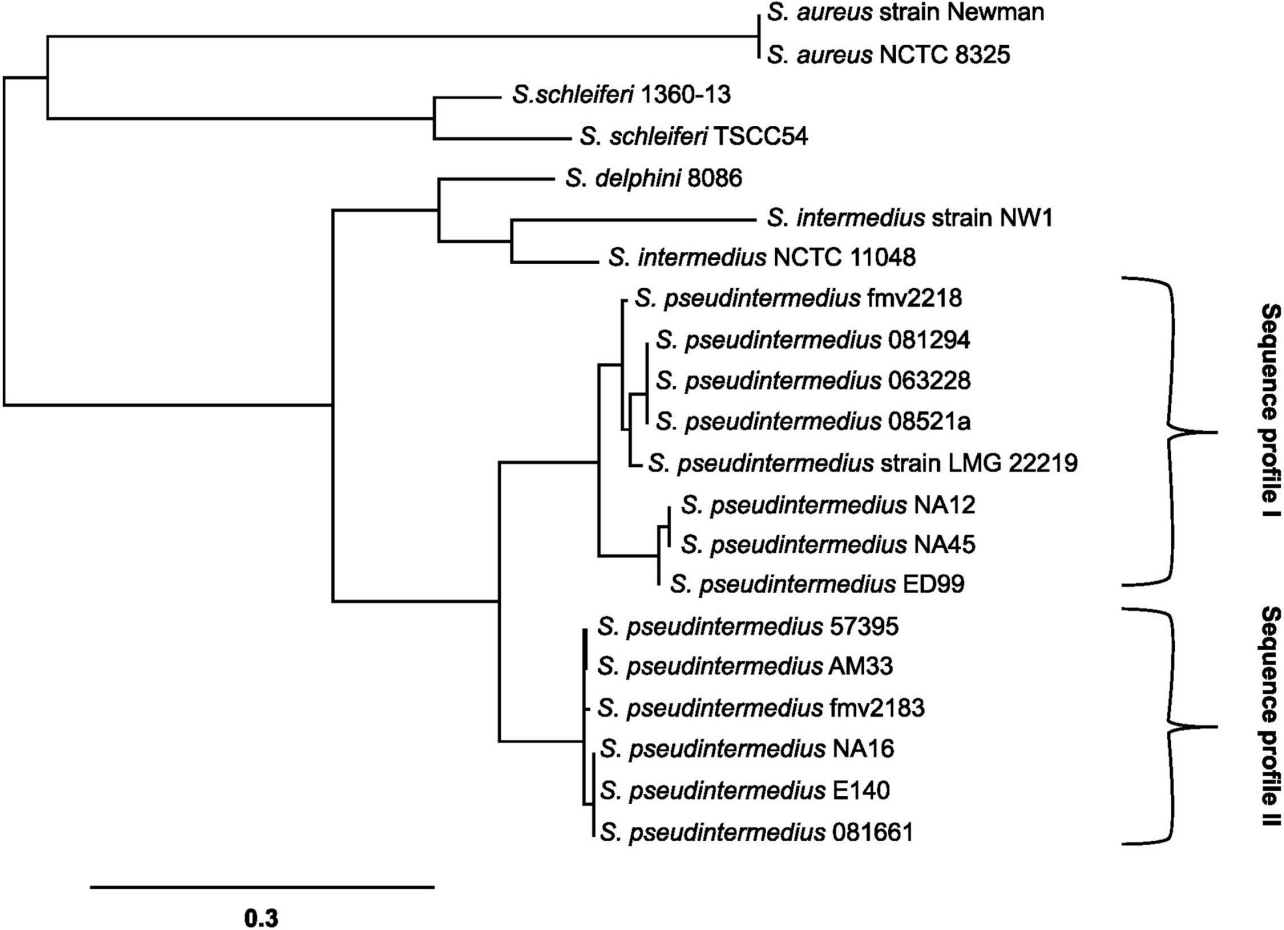
Phylogenetic tree showing the inferred evolutionary relationships among Sbi amino acid sequences from *S*. *aureus*, *S*. *pseudintermedius* and other members of the *Staphylococcus intermedius* group. The scale bar represents a genetic distance equivalent to nucleotide substitutions per 100 residues. *S*. *aureus subsp*. *aureus* strain Newman (ATC72596.1), *S*. *aureus* NCTC 8325(WP_000792564.1), *S*. *pseudintermedius* LMG 22219 (WP_070407297.1), *S*. *pseudintermedius* ED99 (WP_014614922.1), *S*. *intermedius* NCTC 11048 (WP_019169150.1), *S*. *intermedius* NW1 (WP_086428802.1), *S*. *delphini* 8086 (WP_019166019.1), *S*. *schleiferi* 1360-13(WP_050346165.1) and *S*. *schleiferi* TSCC54 (WP_060830457.1) Sbi sequences were obtained from the GenBank database. Braces indicate the two Sbi sequence profiles; sequence profile I, similar to NA45 (CC84) Sbi and sequence profile II, similar to 081661 (ST71) Sbi sequence.

PCR products were sequenced at the University of Tennessee, Molecular Biology Resource Facility using the chain termination method. The BLAST sequence alignment tool (http://www.ncbi.nlm.nih.gov/blast/) and Geneious software (Biomatters) were used to determine nucleotide sequence similarities between *S*. *pseudintermedius* isolates. A phylogentic tree was generated using an online tool (http://www.phylogeny.fr/) comparing Sbi protein from *S*. *aureus* and *S*. *pseudintermedius* (Fig 2) using strains listed in Table 3 and the *S*. *pseudintermedius* type strain, LMG 22219 Sbi sequence was obtained from GenBank WP_070407297.1 [25]. The predicted secondary protein structure was generated with an online tool (https://swissmodel.expasy.org/) and visualized using the PyMol software viewer. The secondary structures of Sbi-I, and Sbi-II were built on PDB code1zxg.1.A [26], and 4npf.2.A [27] protein A templates respectively and Sbi-III and Sbi-IV were built on the PDB code 2jvg.1.A template [28].

### Cloning, expression, and purification of pSbi

Cloning of *pSbi* was carried out using the pETBlue™-2 vector (MilliporeSigma) with full-length *psbi* genes. PCR products from *S*. *pseudintermedius* strains NA45 and 081661 were ligated into the vector, transformed into DH5-alpha *E*.*coli*, and plated on LB agar containing 50μg/ ml ampicillin, 0.1μM IPTG, and 40μg/ml X-gal. *psbi* constructs were expressed in Tuner™ (DE3) pLacI *E*.*coli* competent cells (MilliporeSigma). They were plated on LB agar containing 50 μg/ml ampicillin and 34 μg/ml chloramphenicol (LB amp-cam). Overnight cultures were inoculated into fresh media and grown to an optical density (600 nm) of 0.6. Isopropyl β-D-thiogalactopyranoside (MilliporeSigma) was added to a final concentration of 1 mM and the cells were incubated at 30°C for an additional 4 hrs in a shaking incubator at 225 rpm for induction of protein expression.

For the purification of recombinant protein, bacteria were suspended in 5 ml of solubilization buffer (BugBuster master mix, Millipore Sigma) containing 20μl protease inhibitor (Cocktail Set III, EDTA-Free, Millipore Sigma) and incubated for 30 min at 37°C in a shaking incubator at 225 rpm. Proteins were purified using an immobilized metal ion affinity chromatography column according to the manufacturer’s instructions (PrepEase Ni-TED column,Thermo Fisher Scientific).

### Western blot analysis of pSbi recombinant proteins

Purified recombinant *S*. *pseudintermedius* Sbi proteins (prSbi) were separated on 7.5% polyacrylamide gels and electrophoretically transferred to nitrocellulose membranes (Thermoscientific). Blots were treated overnight with blocking buffer (BB) containing 5% powdered skim milk and 0.05% polyoxyethylene sorbitan monolaurate (Tween 20) in PBS, pH 7.2 at 4°C. Blocked membranes were incubated with horseradish peroxidase (HRP) -conjugated anti-HIS monoclonal antibody (Thermoscientific, 1:2000 dilution) for 1 h at room temperature followed by three washes with TBST buffer (PBS containing 0.05% Tween20). Reactive bands were visualized using 4-chloro-1-naphthol solution (1-Step,Thermo Scientific).

Binding of prSbi to dog IgG was determined using western blots (prepared and blocked as described above) incubated with dog IgG whole molecule (Rockland Antibodies and Assays) diluted 1:1000 and detected with HRP conjugated goat anti-dog IgG (H+L chain, Bethyl), diluted 1:3000.

### ELISA and binding assays

For ELISA and binding assays proteins were diluted in PBS, pH 7.2 and bound to plates (Costar brand 96-well, Fisher Scientific) overnight at 4°C overnight. Unbound protein was removed by washing three times with PBST and ligand, diluted in PBS, was added and incubated for one hour at 37°C. After washing three times with PBST, bound protein was detected using HRP-conjugated reagent, incubated for one hour at 37°C. Plates were washed three times and conjugate visualized using TMB substrate (Thermo Fisher Scientific). Reactions were stopped by adding 50 μl/well of 0.18M sulfuric acid and the optical density measured at 450 nm (BioTek EIA reader).

### Canine IgG, Fc, and Fab binding assays

prSbi proteins derived from NA45 and 081661 were coated onto 96 well plates, reacted with dog IgG (Rockland Antibodies and Assays) and bound IgG was detected using HRP-conjugated goat anti-dog IgG (H+L chain, Bethyl).

To detect IgG Fc and IgG Fab bound to prSbi, canine IgG was digested with immobilized papain using a Fab preparation kit (Thermo Fisher Scientific) as previously described [29]. prSbi (1 μg/ ml) coated plates were incubated with canine Fc, and Fab (beginning concentration 1 μg/ ml then two-fold serially diluted) and detected with HRP conjugated sheep anti-dog IgG heavy chain (1:10,000) (Bethyl).

### prSbi binding to canine IgG

prSbi recombinant proteins from NA45 and 081661 were labeled with sulfo-NHS-LC-biotin (Thermo Scientific) according to the manufacturer's instructions except that a 10-fold molar excess of biotin was used. Dog IgG coated on plates at 1 μg/ ml was incubated with biotinylated prSbi (beginning concentration 1 μg/ ml then two-fold serially diluted) and detected with HRP conjugated streptavidin (1: 10,000) (Thermoscientific).

### Canine, equine, feline, and bovine immunoglobulin binding assays

prSbi protein (1 μg/ ml) was coated on 96 well plates and incubated with canine, equine, feline, or bovine serum (two-fold serially diluted) and bound antibody detected with corresponding HRP conjugated antibodies. These included goat anti-dog IgG (H+L chain, Bethyl), goat anti-horse IgG (Bethyl), goat anti-cat IgG (H+L) (Kirkegaard and Perry laboratories), sheep anti-bovine IgG (Bethyl), goat anti-dog IgM (mμ) (Kirkegaard and Perry laboratories), goat anti-horse IgM (Bethyl), goat anti- cat IgM (mμ) (Kirkegaard and Perry laboratories), and sheep anti-bovine IgM-mμ (Bethyl) (1:10,000 dilution). To exclude cross reactivity of canine IgG with anti-dog IgM, canine IgG (1 μg/ ml) coated plates were incubated with HRP-conjugated goat anti-dog IgG (H+L chain, Bethyl), or peroxidase labeled affinity purified goat anti- dog IgM (Kirkegaard and Perry laboratories) (1:10,000 dilution).

### Complement mediated hemolysis assay

A hemolysis assay was performed to determine the ability of prSbi to inhibit complement activity. Bovine erythrocytes were sensitized to complement by incubation with rabbit IgG fraction anti-bovine red blood cells (1:25 dilution, ICN), for 30 min at 37°C with gentle mixing. Following preincubation of 5 or 10% dog serum with 600, 900, or 1,500 ng/ml of prSbi from NA45 or 081661 for 30 min at 37°C with gentle shaking (100 rpm), 100 μl of sensitized bovine RBCs were added and further incubated for 30 min at 37°C with gentle shaking (100 rpm). After centrifugation (5 min, 5,000 rpm) the absorbance of the supernatant was measured at 405 nm and compared with negative (heat inactivated serum) and positive (5% and 10% normal dog serum) controls. Complement activity percentage was quantified from the absorbance at 405 nm using the equation: (sample—negative control)/(positive control—negative control × 100%) = complement activity [8].

### Binding of biotinlylated prSbi to canine B cells

Mononuclear cells were separated from canine peripheral blood using cell separation media (HistoPaque1077, Sigma) according to the manufacturer’s protocol. Experimental protocols for obtaining samples of dog blood were reviewed and approved by the University of Tennessee Institutional Animal Care and Use Committee (IACUC) under protocol 2474–0716. Canine B cells were labeled with phycoerythrin (PE) conjugated mouse anti-canine CD21 (Bio-Rad) then incubated with biotinylated prSbi (3 μg/ml) at 37°C for 30 min with gentle shaking (100rpm), washed with PBS and incubated with FITC-conjugated avidin (1:200 dilution, Sigma). Cells were washed with PBS and B cell surface-bound biotinylated prSbi protein was analyzed by flow cytometry (Applied Biosystems Attune flow cytometer, Thermo Fisher Scientific). Lymphocytes were gated based on forward and side scatter and B cells within this region further identified with PE anti-CD21, were analyzed for FITC-conjugated avidin bound to biotin-prSbi by determining the fluorescence of 10,000 B cells for each sample. Mononuclear cells incubated with FITC-conjugated avidin and without prSbi served as negative controls.

### Statistical analysis

Data were analyzed using factorial ANOVA with the response variables being measurements of prSbi biotinylated protein binding to IgG, complement-mediated_haemolysis_assay, prSbi binding to IgG, Fab, and Fc domains, and prSbi binding to IgG and IgM of different animal species and independent variables being dilutions, and the two different recombinant proteins. Ranked transformation was applied if diagnostic analysis exhibited violation of normality and equal variance assumptions. Post hoc multiple comparisons among treatment levels were conducted with Tukey’s adjustment. Data represent mean values from three independent experiments with each plot in the graph representing the average of duplicate wells, error bars indicate standard deviations. Statistical significance was identified at the level of 0.05. All analysis was conducted using PROC GLM in SAS 9.4 TS1M3 for Windows (SAS institute Inc.).

## Results

### Amplification and sequencing of *sbi* from *S*.*pseudintermedius*

The putative *S*. *pseudintermedius sbi* gene was amplified by PCR from 13 strains of *S*. *pseudintermedius* and yielded a product of the expected size of approximately 1,500 bp. Four additional isolates were negative for *psbi* using 3 different primer sets (Table 3). DNA sequencing of the 13 isolates showed two *psbi* sequence profiles. Sequence profile I was homologous to strains NA45 and NA12 (both CC84 in the study) and included all CC68 isolates except FMV9/08, which was PCR negative. Sequence profile II was homologous to strain 081661 (ST71) and included all three ST71s (Fig 2). The *psbi* open reading frame encodes a protein with a predicted molecular mass of 55,600 and 55,050 daltons from strains NA45 and 081661, respectively. It corresponds to bases 2809021 to 2810517 and 2698509 to 2699996 in the genomes of *S*. *pseudintermedius* strains NA45 and 081661 (GenBank CP016072.1 and CP016073.1) [24]. The sequence alignment of amino acid residues corresponding to each Sbi domain (I-IV) is indicated in S2 Fig. The predicted pSbi domains and molecular structures are presented in Figs 1 and S1. The predicted pSbi protein contains two IgG binding domains with 64% identity to Sbi I and Sbi II domains of *S*. *aureus* NCTC8325 (GenBank WP_000792564.1) and 45% identity to Spa-E and Spa-D domains of *S*. *aureus* Newman (GenBank BAF66327.1) protein A [30]. By comparison, *S*. *aureus* NCTC8325 IgG binding domains of Sbi protein have only 30% identity to Spa-E and Spa-D domains of the same *S*. *aureus* strain. The Fab binding region of Sbi contains two aspartic acid residues in the Spa-E and Spa-D domains of *S*. *aureus* Newman compared to *S*. *pseudintermedius* pSbi which has only one aspartic acid in the corresponding region, and *S*. *aureus* NCTC8325 Sbi lacks both aspartic acid residues. pSbi also contains two complement binding domains with 53% and 17.39% identity to the Sbi-IV domain of *S*.*aureus* NCTC 8325 Sbi protein (GenBank WP_000792564.1).

**Table 3:** Amplification of the *sbi* gene from three different primer sets.

Strain	Product from Primer Set		
	Primer NA45	Primer 081661	Primer 063228
NA45	POS	NEG	NEG
NA12	POS	NEG	NEG
E140	POS	NEG	NEG
81661	NEG	POS	NEG
E141	NEG	POS	NEG
NA16	NEG	POS	NEG
57395	NEG	POS	NEG
FMV2183-10	NEG	POS	NEG
Am33	NEG	POS	NEG
63228	NEG	NEG	POS
0821a	NEG	NEG	POS
81294	NEG	NEG	POS
FMV2218-10	NEG	NEG	POS
FMV5699/07	NEG	NEG	NEG
FMV9/08	NEG	NEG	NEG
NA73	NEG	NEG	NEG
NA74	NEG	NEG	NEG

### Sbi recombinant protein binding to immunoglobulins

**Fig 3:**
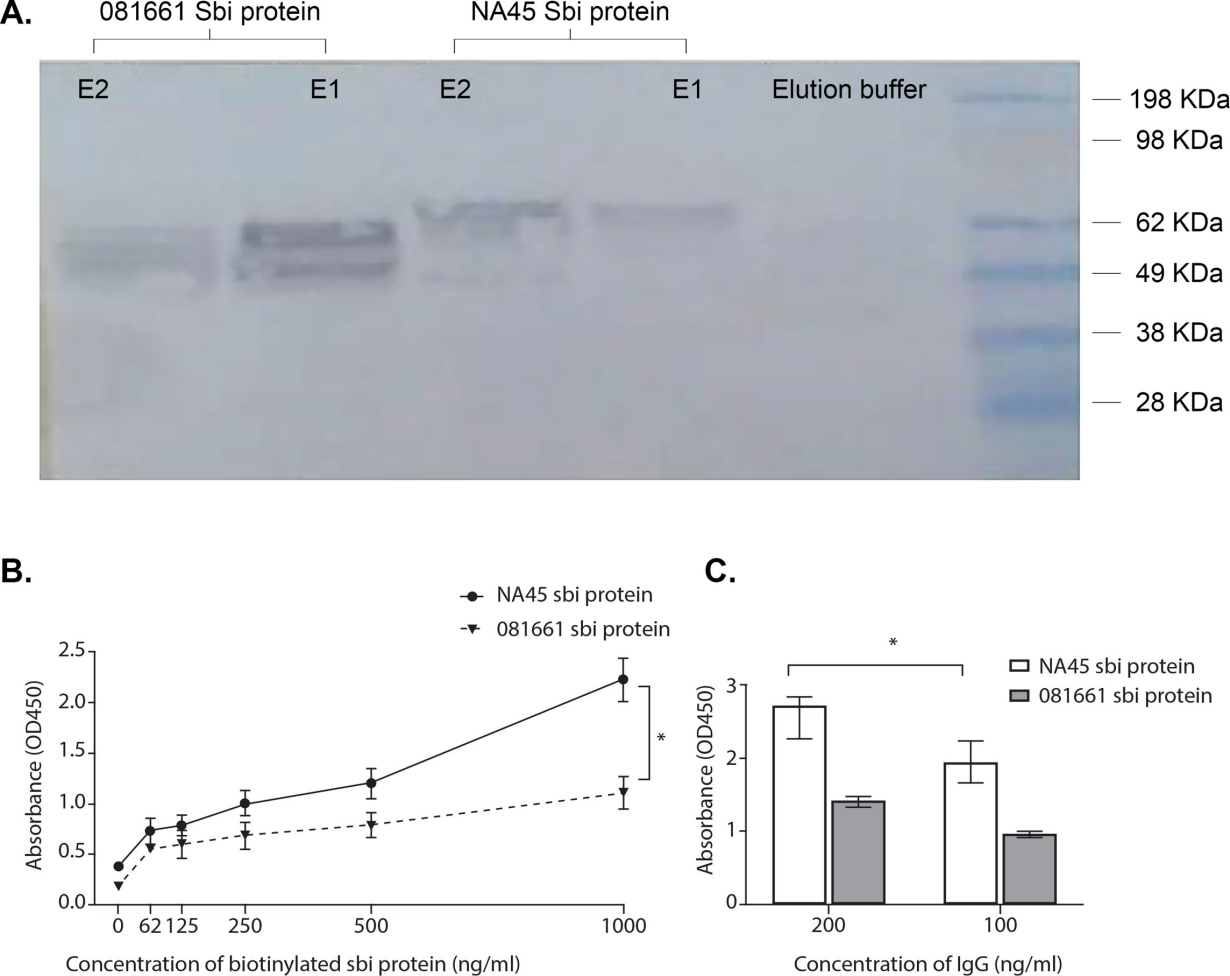
Canine IgG bound to Sbi recombinant proteins of strains NA45 and 081661. (A) Canine IgG bound to prSbi recombinant proteins detected by western immunoblot using (HRP)-conjugated goat anti-dog IgG. prSbi bound canine IgG is shown in comparison to elution buffer. Immunoreactive bands correspond to 62KDa and 60KDa for strains NA45 and 081661, respectively. (B) Binding of canine IgG to biotinylated prSbi. Biotinylated Sbi recombinant proteins bound to canine IgG coated on ELISA plates. prSbi from strain NA45 bound IgG at a significantly higher level than that of strain 081661 (*p <0.0001). (C) Binding of canine IgG to unlabeled prSbi. Sbi recombinant protein of strain NA45 bound IgG at a higher level than that of strain 081661 (*p <0.0001) as determined in an ELISA with HRP-anti-canine IgG. All concentrations were significantly different from the negative control (buffer) (p<0.05).

The immunoglobulin-binding properties of prSbi resolved by SDS-PAGE were analysed in western blots. Immunoreactive bands of 62KDa and 60KDa were detected from *S*.*pseudintermedius* strains NA45 and 081661 Sbi proteins, respectively. These mass estimates include the putative Sbi proteins with their signal peptides and the HSV and HIS C-terminal tags from pet blue-2 plasmid (Fig 3A).

**Fig 4:**
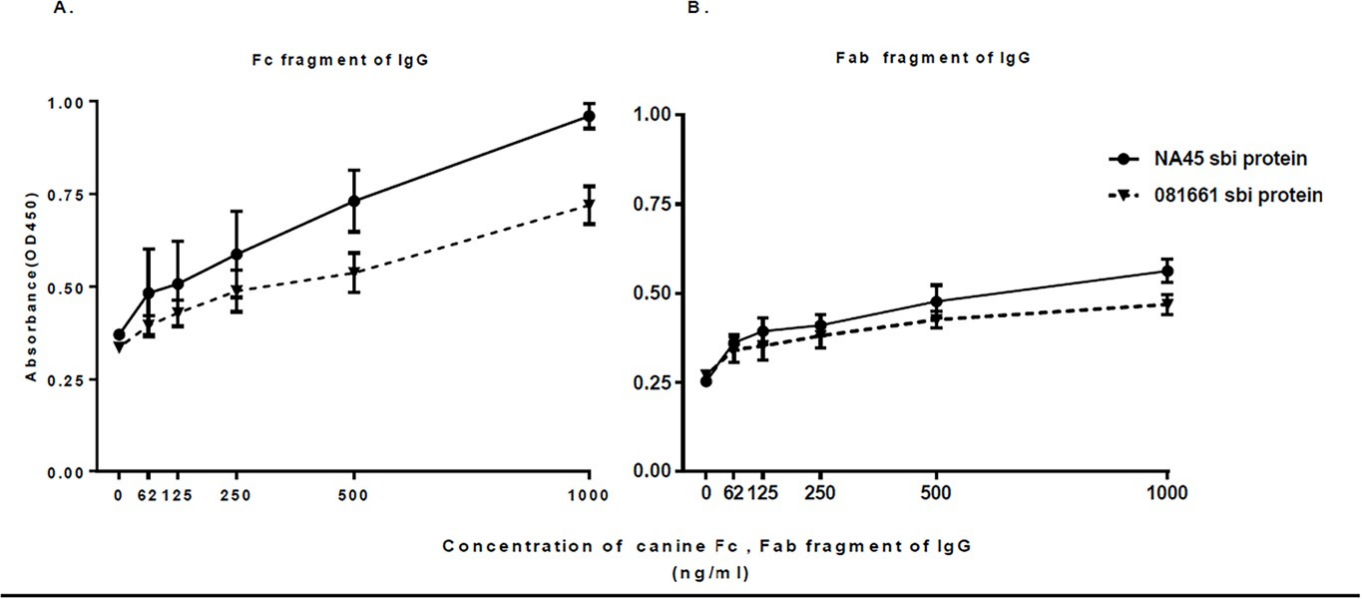
The association of immobilized Sbi recombinant protein with IgG fractions determined by ELISA. Sbi bound Fc (panel A) and Fab (panel B) fragments of canine IgG. Significantly more binding occurred with prSbi from NA45 than from 081661 (p = 0.0271). Reactivity with all concentrations was significantly different compared to the negative control (buffer) (p<0.05) except for 62ng/ml and125ng/ml (Ns). Sbi proteins of strains NA45 and 081661 bound canine, equine, and feline IgG p<0.0001 but did not significantly bind bovine IgG compared with the buffer control. The binding of Sbi proteins with canine IgG was higher than with feline IgG and equine IgG p<0.05 (Fig 5A).

Biotin-labeled prSbi proteins from both NA45 and 081661 bound canine IgG (Fig 3B). To exclude non-specific binding of prSbi via its biotin-label, binding of unlabeled prSbi to canine IgG was tested using ELISA. Canine IgG bound to both NA45 and 081661 prSbi (Fig 3C). Significantly higher binding occurred with NA45 prSbi (sequence profile I) compared with 081661 prSbi protein (sequence profile II) (p <0.0001). prSbi bound both Fc and Fab domains of canine IgG as determined by ELISA. NA45 prSbi bound significantly more Fc (Fig 4A) and Fab (Fig 4B) than 081661 prSbi (p = 0.0271) and both had greater binding to Fc than Fab fragments.

**Fig 5:**
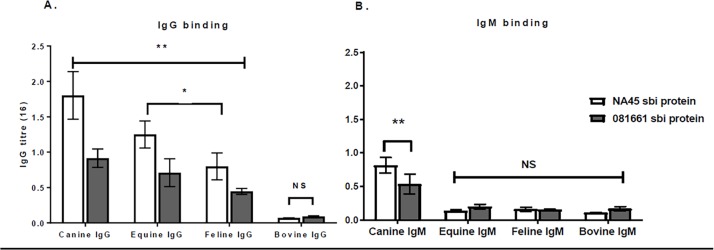
prSbi binding to IgG, IgM and IgG from various animal species. (A) prSbi of NA45 and 081661 binding to IgG was not significantly different p 0.0541. They bound canine, equine, and feline IgG ** (p<0.0001) and did not significantly bind bovine IgG (NS) in comparison with negative controls. The binding of prSbi proteins with feline IgG and equine IgG was lower than canine IgG *(p<0.05). (B) Binding of prSbi to IgM. prSbi from NA45 and 081661 bound significantly different amounts of IgM p = 0.045. They had significant reactivity with canine IgM **p<0.0001 and not with bovine, equine, and feline IgM (NS).

prSbi of strains NA45 and 081661 bound canine IgM (p<0.0001) and did not significantly bind bovine, equine, or feline IgM (Fig 5B). Higher binding occurred with prSbi from strain NA45 (sequence profile I), than with that of Sbi protein from strain 081661 (sequence profile II) (p = 0.045).

### Sbi recombinant protein bound to canine B cells

**Fig 6:**
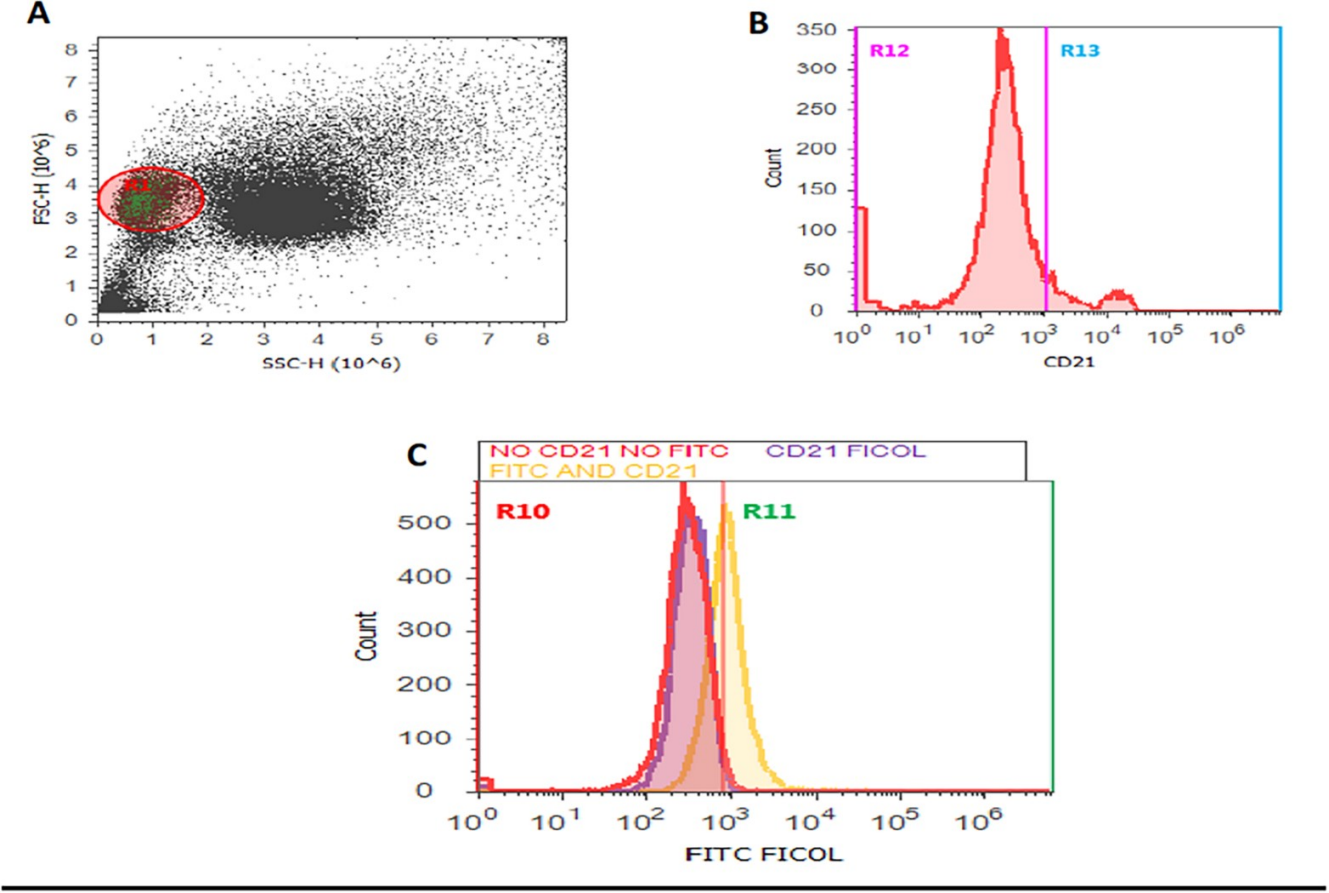
Binding of biotinlylated prSbi to canine B cells measured using flow cytometry. (A) The lymphocyte population was identified using forward and side scatter. (B) B cells labeled with anti-CD21 (Region R13) were selected for analysis of prSbi binding. (C) Binding of biotinylated prSbi with B cells was measured by the detection of FITC-avidin conjugate in region R11 (yellow peak) and compared with negative controls consisting of PE anti-CD21 and FITC-avidin without biotin (red peak) and PE anti-CD21 alone (violet peak).

Using flow cytometry and gating on B cells from canine blood using forward scatter and side scatter characteristics (Fig 6A) as well as anti-CD21 (Fig 6B), strong binding of biotinlylated Sbi protein to canine B cells was detected in comparison with buffer control that lacked Sbi (p<0.05)(Fig 6C).

### prSbi inhibits complement function

**Fig 7:**
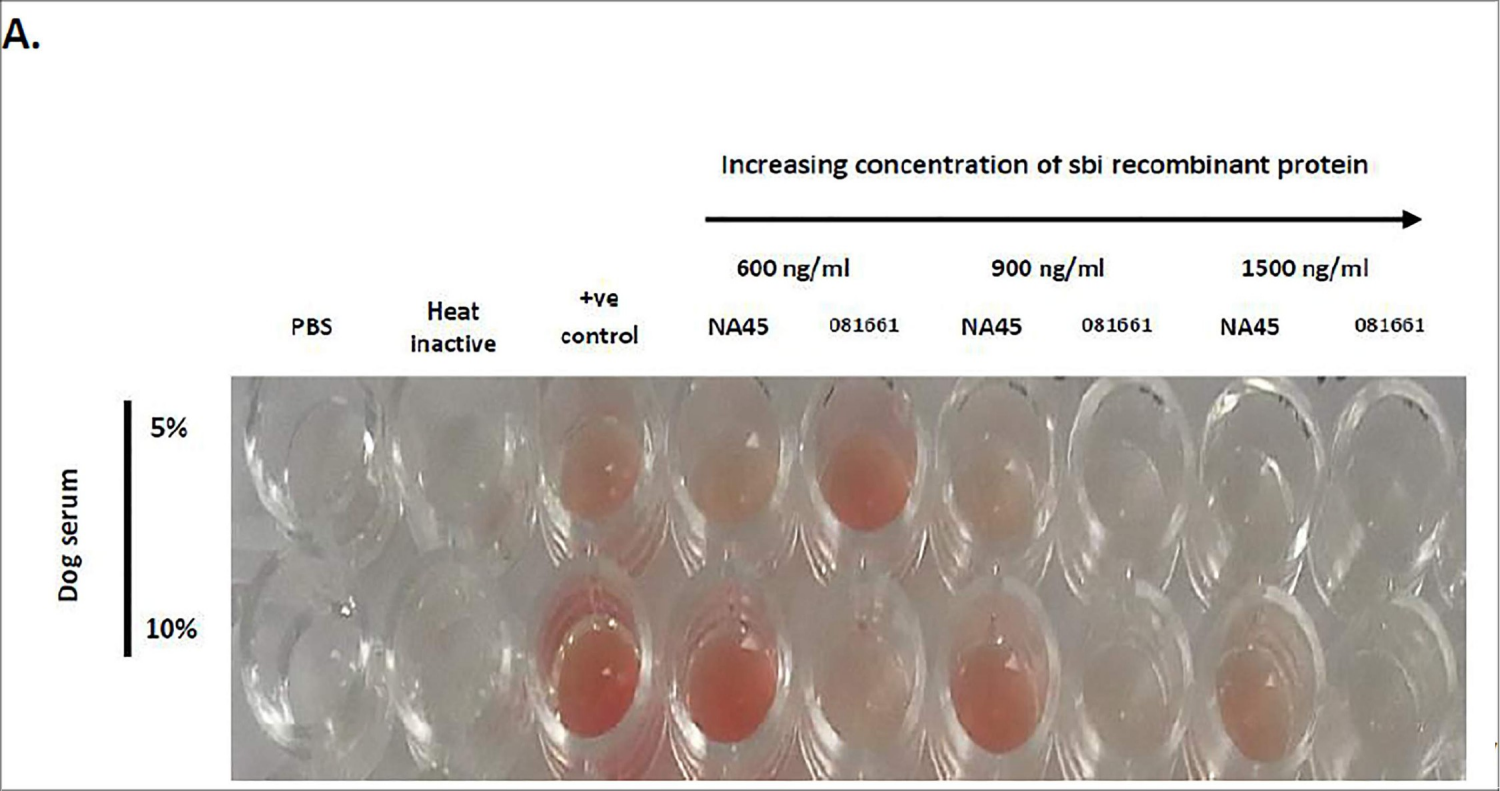
prSbi inhibits complement activity. Hemolysis of sensitized bovine erythrocytes was assayed in the presence of increasing concentrations of prSbi from NA45 and 081661. prSbi from of 081661 was significantly more effective than NA45 prSbi (p <0.0001). All concentrations of prSbi from NA45 were different from the negative controls *p<0.05 except at 1500ng/ml whereas none of the concentrations of 081661 prSbi differed from the negative control (Ns).

prSbi inhibited hemolysis of sensitized bovine erythrocytes. The complement inhibition activity of prSbi from *S*. *pseudintermedius* strain 081661 was significantly higher than that of Sbi protein from strain NA45 (p <0.0001). All concentrations tested of prSbi from strain 081661 inhibited complement mediated hemolysis whereas only the highest concentration of NA45 prSbi protein (1500ng/ml) significantly inhibited hemolysis (Fig 7).

## Discussion

Staphylococci use an array of virulence factors to overcome their hosts’ immune systems including immunoglobulin binding proteins [31]. Among these proteins the antibody binding properties and superantigenic effect of protein A has been well characterized in *S*. *aureus* and *S*. *pseudintermedius* [29, 32]. Similarly, *S*. *aureus* Sbi binds IgG [10] and complement C3 [8, 33] and *S*. *pseudintermedius* coagulase has been shown to bind C3 and immunoglobulin [34]. However, a *S*. *pseudintermedius* Sbi ortholog has not been studied and the difficulty of selectively knocking out genes in *S*. *pseudintermedius* makes the study of interactions of Sbi on bacteria with ligands challenging. A *S*. *pseudintermedius* gene is routinely annotated as Sbi based on limited sequence similarity, but little is known about the activity of this putative Sbi, whether it serves as a virulence factor with activity similar to *S*. *aureus* and if it is adapted to *S*. *pseudintermedius’* hosts. *S*. *aureus* primarily infect humans and *S*. *pseudintermedius* transiently infect humans but primarily cause disease in dogs, cats and to a lesser extent horses [5, 35, 36]. The purpose of this study was to determine the structure and functional characteristics of pSbi and its interaction with the immune system of its predominant host, the dog, in comparison with other mammalian species. Recombinant Sbi was used to determine the activity of this protein without the confounding effects of other surface proteins known to bind immunoglobulins and complement components as well as potentially other as yet unidentified surface proteins with similar binding activity.

Although they share only about 40 to 44% similarity, Sbi proteins of *S*. *aureus* and *S*. *pseudintermedius* have the same general domain structures and, as shown in this study, are predicted to form similar α-helices. However, regions III, IV, Wr and Y of pSbi are somewhat larger and, consequently, overall pSbi contains approximately 59 more amino acids (depending on strains) than Sbi. Aspartic acid has been identified as being important for immunoglobulin binding and in *S*. *aureus* Newman protein A, the Fab binding region contains aspartic acid residues at positions 36 and 37. In comparison, they are absent in *S*. *aureus* Newman Sbi protein [37] and one corresponding aspartic acid is present in pSbi.

pSbi binds to canine, equine, and feline IgG but not significantly to bovine IgG. This binding pattern corresponds with the ability of *S*. *pseudintermedius* to naturally infect dogs, cats, and horses whereas it is rarely isolated from cattle [38]. The binding of pSbi to Fab and Fc fragments of canine IgG as well as B cells is in agreement with previous findings for *S*.*aureus* Sbi protein that showed the association of immobilized Sbi with Fc or F(ab)2 fragments of human IgG [15]. The immunoglobulin binding activity of pSbi is not restricted to canine IgG as pSbi also bound canine IgM in this study. There is evidence to suggest that *S*. *aureus* Sbi also binds IgM although this has not been examined in detail [9].

Microbes are often effectively killed and eliminated by the complement system by deposition of C3b onto their surface, which facilitates opsonization, elimination of the microbe by phagocytosis, and lysis by formation of the membrane attack complex (terminal complement complex) [39]. *S*. *aureus* Sbi inhibits initiation of the classical complement pathway by interfering with C1q binding to IgG [9, 15]. Sbi also manipulates the molecular link between innate and adaptive immune responses by inhibiting complement receptor 2 (also designated CD21) interaction with C3 (antigen-associated C3dg or iC3b) [10, 13, 15]. The three-helix bundle fold of domains III and IV found in *S*. *pseudintermedius* are similar to *S*. *aureus* Sbi in which they are associated with complement inhibition [28]. In this study, we demonstrate that *S*. *pseudintermedius* pSbi protein is a potent complement inhibitor that blocks the hemolytic activity of dog serum on sensitized bovine erythrocytes in a concentration dependent manner, although more so with pSbi from 081661 than NA45.

Sbi sequences from different *S*.*aureus* strains show high levels of conservation in both nucleotide and protein sequences [10]. The predicted *S*. *pseudintermedius* Sbi proteins diverge into two major groups that correlate with multilocus sequence type. Sequence profile I occurs with isolates representing clonal complexes CC68 and CC84, associated with methicillin resistant strains in the United States [6]. Sequence profile II was found in ST71 that occur in Europe, the United States and are common throughout the world. It is interesting to note that pSbi from sequence profile I had relatively higher binding to IgG, Fc and Fab, but lower inhibition of complement compared with profile II pSbi. The major difference between the proteins is in domain IV at the end of the alpha 2 region and the area between alpha 2 and alpha three. In this region NA45 Sbi contains four more amino acids than 081661. The effect of this difference warrants further investigation. The failure of four out of seventeen *S*. *pseudintermedius* isolates to produce a *Sbi* PCR product suggests that the protein may be absent in some strains. This may also be explained by a lack of homology with PCR primers and the possibility that additional genotypes of Sbi exist in *S*. *pseudintermedius* cannot be excluded. However, it has been noted that Sbi is found in most, but not all strains of *S*. *aureus* [25, 40].

## Conclusion

The gene examined in this study encodes a novel *S*. *pseudintermedius* Sbi protein that displays the immunoglobulin binding and complement inhibitory ability of *S*. *pseudintermedius* strains representative of major clonal lineages, CC71, CC68 and CC84. Sbi has the ability to bind canine IgG and IgM, binds IgG from a wide range of animal species including dog, cat and horse and inhibits complement activity. Moreover, the ability of this protein to bind to canine B cells suggests that it may manipulate the adaptive immune response. Therefore, pSbi may be a promising target for vaccine development for treatment or prevention of disease caused by *S*. *pseudintermedius*.

## Supporting information

S1 Fig Secondary structure of S. pseudintermedius NA45 Sbi domains I-IV. The α-helices are colored blue and numbered and the beta strands are presented in pink.
(PDF)

S2 Fig Sequence alignment of *S*.*pseudintermedius* Sbi domains I-IV. The secondary α-helices of Sbi I-IV are shown. White, red, gray,and blue shading indicate 100%, 80–100%, 60–80%, and less than 60% similarity between sequences respectively. (A)Comparison of amino acid sequences of IgG binding domains of Sbi protein from *S*. *aureus*, *S*. *pseudintermedius* and *S*. *aureus* Spa protein. Two glutamine (Q) residues represent Fc binding regions, and two aspartic acid (D) residues representing Fab binding regions are highlighted by gray rectangles. (B) Comparison of amino acid sequences of complement C3 binding domain (Sbi-IV) of *S*. *aureus* and *S*. *pseudintermedius* Sbi protein. Arginine (R) and asparagine (N) amino acid residues representing complement binding sites are highlighted by gray rectangles.
(PDF)
