# Supplementary material for: Staphylococcus pseudintermedius Sbi paralogs inhibit complement and bind IgM, IgG Fc and Fab
Source: PLoS One. 2019 Jul 23;14(7):e0219817. doi: 10.1371/journal.pone.0219817 (PMC6650138; doi:10.1371/journal.pone.0219817)
Supplement: S1 Fig — The α-helices are colored blue and numbered and the beta strands are presented in pink. (PDF) [file pone.0219817.s001.pdf]

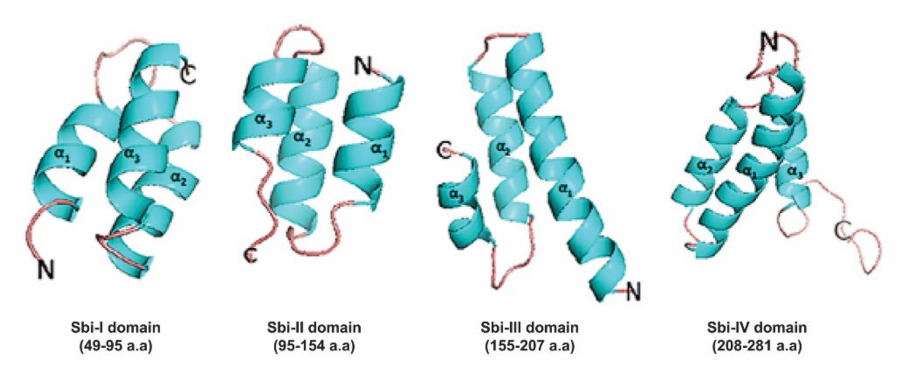

S1 fig. Secondary structure of S. pseudintermedius NA45 Sbi domains I-IV. The α-helices are colored blue and numbered and the beta strands are presented in pink.
