# Supplementary material for: Staphylococcus pseudintermedius Sbi paralogs inhibit complement and bind IgM, IgG Fc and Fab
Source: PLoS One. 2019 Jul 23;14(7):e0219817. doi: 10.1371/journal.pone.0219817 (PMC6650138; doi:10.1371/journal.pone.0219817)
Supplement: S2 Fig — The secondary α-helices of Sbi I-IV are shown. White, red, gray,and blue shading indicate 100%, 80–100%, 60–80%, and less than 60% similarity between sequences respectively. (A)Comparison of amino acid sequences of IgG binding domains of Sbi protein from S. aureus, S. pseudintermedius and S. aureus Spa protein. Two glutamine (Q) residues represent Fc binding regions, and two aspartic acid (D) residues representing Fab binding regions are highlighted by gray rectangles. (B) Comparison of amino acid sequences of complement C3 binding domain (Sbi-IV) of S. aureus and S. pseudintermedius Sbi protein. Arginine (R) and asparagine (N) amino acid residues representing complement binding sites are highlighted by gray rectangles. (PDF) [file pone.0219817.s002.pdf]

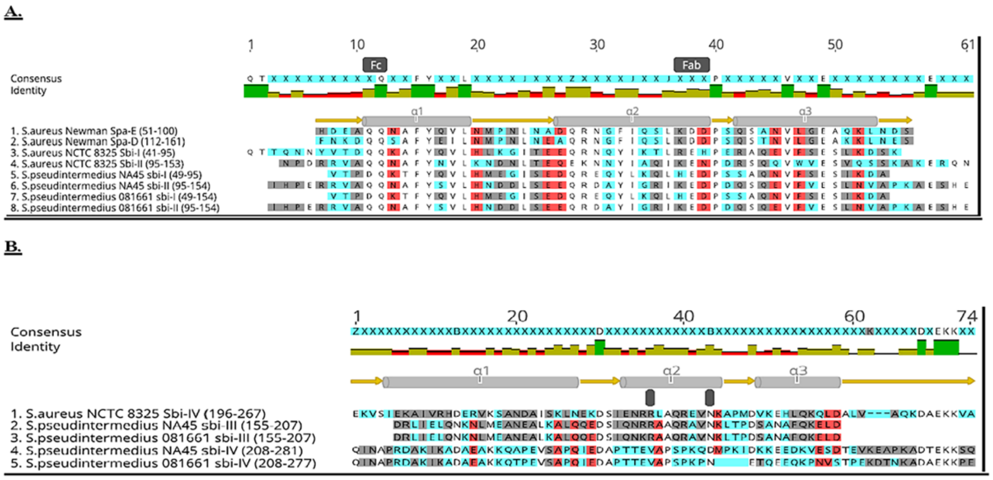

S2 Fig. Sequence alignment of S.pseudintermedius Sbi domains I-IV. The secondary α-helices of Sbi I-IV are shown. White, red, gray,and blue shading indicate 100%, 80-100%, 60-80%, and less than 60% similarity between sequences respectively. (A)Comparison of amino acid sequences of IgG binding domains of Sbi protein from S. aureus , S. pseudintermedius and S. aureus Spa protein . Two glutamine (Q) residues represent Fc binding regions, and two aspartic acid (D) residues representing Fab binding regions are highlighted by gray rectangles. (B) Comparison of amino acid sequences of complement C3 binding domain (Sbi-IV) of S. aureus and S. pseudintermedius Sbi protein. Arginine (R) and asparagine (N) amino acid residues representing complement binding sites are highlighted by gray rectangles.
